# Reviewed article: Silva RES, Lima EA, Silva AVL, Sousa JF, Oliveira EAR, Silva DMC, Carvalho MF, Carvalho RBN. Individual and contextual characteristics associated with concurrent risk factors among adolescents: a multilevel cross-sectional study based on the 2019 Brazilian National Health Survey. Epidemiol Serv Saude. 2025:34;e20240453.

**DOI:** 10.1590/S2237-96222025v34e20240453.b

**Published:** 2025-09-08

**Authors:** Dartagnan Pinto Guedes

**Affiliations:** Universidade Estadual do Norte do Paraná, Centro de Ciências da Saúde

**Table:** 

Rating	Excellent	Good	Average	Below average	Poor
Interest		✓			
Quality		✓			
Originality		✓			
Overall		✓			

**Table:** 

Questionnaire	Yes	No	Not applicable
Does the manuscript contain new and significant information to justify publication?	✓		
Does the Abstract (Summary) clearly and accurately describe the content of the article?	✓		
Is the problem significant and concisely stated?	✓		
Are the methods described comprehensively?	✓		
Are the interpretations and conclusions justified by the results?	✓		
Is adequate reference made to other work in the field?	✓		

## Manuscript Structure

Length of article is: Adequate

Number of tables is: Adequate

Number of figures is: Adequate

Please state any conflict(s) of interest that you have in relation to the review of this paper (state “none” if this is not applicable): None

## Comments

The manuscript provides the findings of a study conducted with the objective to assess the simultaneity of risk behaviors for chronic non-communicable diseases and associate them with individual and contextual characteristics in Brazilian adolescents. The theoretical basis is well founded and justifies the study. The bibliographic references are current and relevant to the subject of study. The methods used are sufficiently described, appropriate and consistent with the study proposal. The results are presented in an attractive manner, and the discussion of the findings show good prospects to the knowledge area. The conclusions are consistent with presented evidence and arguments. The findings of the study have good prospects to offer important contributions to the knowledge area, and offers important support for the design and conduct of future studies.

